# Menthol Tobacco Product Use Among US Middle and High School Students, National Youth Tobacco Survey, 2023–2024

**DOI:** 10.5888/pcd23.250427

**Published:** 2026-08-06

**Authors:** Katherine A. Moran, Philip E. Rosenbaum, Christina Meyers, Monica Cornelius, Margaret Mahoney, Michael Sawdey, Samantha N. Cwalina, Livia Navon

**Affiliations:** 1 Office on Smoking and Health, National Center for Chronic Disease Prevention and Health Promotion, Centers for Disease Control and Prevention, Atlanta, Georgia; 2 Katmai Government Services, LLC, Orlando, Florida; 3 Division of Population Health Science, Office of Science, Center for Tobacco Products, US Food and Drug Administration, Silver Spring, Maryland

## Abstract

**Introduction:**

Most tobacco use begins during adolescence. We used National Youth Tobacco Survey (NYTS) data from 2023 and 2024 to examine menthol tobacco product use among US middle and high school students.

**Methods:**

We used pooled data from the 2023 and 2024 NYTS (n = 51,930) to calculate nationally representative prevalence estimates for past 30-day menthol and nonmenthol tobacco product use, stratified by sociodemographic and mental health indicators. We used χ^2^ tests with Benjamini–Hochberg adjustments to compare behaviors.

**Results:**

During 2023 and 2024, 22.1% of students who currently used tobacco (representing 520,000 students) used menthol products. Overall, menthol products were used by 40.0% of students who smoked cigarettes, 18.0% who used e-cigarettes, and 11.5% who smoked cigars. Menthol tobacco use was significantly higher among males (vs females) (26.1% vs 18.3%), high schoolers (vs middle schoolers) (23.9% vs 17.0%), and those with moderate to severe anxiety or depression (vs no or mild symptoms) (25.0% vs 18.8%). By race and ethnicity, use ranged from 12.0% (non-Hispanic Black) to 28.6% (non-Hispanic multiracial). Compared with students who used nonmenthol products, a higher proportion of those who used menthol cigarettes or e-cigarettes reported younger age at first use of tobacco (<11 y) (42.5% vs 26.8% and 19.9% vs 13.2%, respectively), use on 20 to 30 of the past 30 days (38.2% vs 20.2% and 53.4% vs 33.2%, respectively), and fewer past-year smoking quit attempts (49.7% vs 63.2%, respectively).

**Conclusion:**

More than 500,000 US middle and high school students used menthol products; prevalence varied by sociodemographic and mental health indicators. Menthol tobacco use correlates with earlier initiation, higher frequency of use, and reduced past-year quit attempts. Additional efforts to reduce access to menthol products among young people may reduce tobacco use in this population.

**Summary**

**What is already known on this topic?**

Menthol reduces the harshness of tobacco and increases appeal and addiction potential among young people. Menthol tobacco products are disproportionately marketed to certain populations; use varies by sociodemographic and mental health factors.

**What is added by this report?**

More than 500,000 US middle and high school students used menthol tobacco products in 2023 and 2024. Compared with those using nonmenthol products, students using menthol products started at younger ages, used more frequently, and — among cigarette smokers — made fewer quit attempts. Menthol tobacco product use also differed by mental health status.

**What are the implications for public health practice?**

Strengthening policies that limit access to menthol tobacco products may reduce initiation and dependence among young people, including groups with disproportionate use.

## Introduction

Most tobacco use begins during adolescence (1). The addition of menthol, a chemical additive, produces a cooling sensation that masks the harshness of tobacco products, making these flavored products more appealing to children, adolescents, and young adults. Menthol can also enhance the effect of nicotine on the brain, increasing nicotine dependence and making cessation more difficult (2,3). Additionally, emerging evidence suggests that menthol in tobacco products may provide additional sensory and pharmacologic effects, potentially contributing to higher rates of anxiety and depression among people using these products (3,4). The public health importance of menthol in tobacco products is compounded by targeted industry marketing strategies. For decades, tobacco companies have localized retail advertisements and price promotions for menthol cigarettes in neighborhoods with higher proportions of young people and racial and ethnic minority residents (2,5,6). Consequently, menthol tobacco use is not uniform across populations. Studies indicate that non-Hispanic Black and Hispanic middle and high school students and young adults with mental health conditions have a disproportionate use of menthol tobacco products (4,7). However, recent data on the evolving landscape of e-cigarette and combustible tobacco use among young people are limited, particularly data analyzed by product type.

Given the dynamic nature of tobacco trends among children, adolescents, and young adults, updated evidence is essential to inform prevention and intervention strategies. This study examined pooled data from the 2023 and 2024 National Youth Tobacco Survey (NYTS) to update prevalence estimates of menthol tobacco use among middle and high school students. We examined differences in use of specific menthol tobacco products by sociodemographic characteristics and mental health status, indicators that are often linked to higher tobacco use. Additionally, we analyzed tobacco-related behaviors, including age of initiation and frequency of use, to compare students using menthol cigarettes, cigars, and e-cigarettes with students using nonmenthol products. These findings can guide public health policies aimed at reducing tobacco initiation and use among young people, including among populations with disproportionate tobacco use.

## Methods

### Data collection

The NYTS is a nationally representative, annual, cross-sectional survey of US students attending public or private middle (grades 6–8) and high (grades 9–12) schools. Students who complete the survey range in age from 9 to 19 years. We combined data from the 2023 and 2024 NYTS (n = 51,930) to allow for stratification of menthol tobacco use by demographic variables with smaller populations. In 2023, 22,069 students from 179 schools participated with an overall response rate of 30.5% (8). In 2024, 29,861 students from 283 schools participated with an overall response rate of 33.4% (9).

### Data analysis

We calculated weighted prevalence estimates, 95% CIs, and weighted population totals of menthol tobacco use among all students and among students who currently used (ie, used on ≥1 day during the past 30 days) specific tobacco products (e-cigarettes, cigarettes, or cigars) and for 2 composite categories of use: any tobacco product use (cigarettes, cigars, smokeless tobacco [chewing tobacco, snuff, or dip], e-cigarettes, hookah, pipe tobacco, snus, oral tobacco, bidis, heated tobacco, or nicotine pouches) and any combustible product use (cigarettes, cigars, hookah, pipe tobacco, or bidis). We calculated product-specific population totals to capture the prevalence of menthol tobacco use. This level of detail is necessary to inform product-specific policy efforts and to prioritize resource allocation, particularly because patterns of tobacco use among children and adolescents differ from patterns among adults (2). We calculated the prevalence of menthol tobacco use using methods similar to those used in previously published studies using the NYTS (8–10).

We stratified prevalence estimates by sociodemographic characteristics and mental health status. Sociodemographic variables were sex (male or female), school level (middle or high school), and race and ethnicity (Hispanic or Latino [Hispanic], non-Hispanic American Indian/Alaska Native, non-Hispanic Asian, non-Hispanic Black or African American, non-Hispanic Native Hawaiian or Other Pacific Islander, non-Hispanic White, and non-Hispanic multiracial). The NYTS uses the 4-question Patient Health Questionnaire-4 to identify students experiencing symptoms of anxiety, depression, or both (11); we used responses to determine mental health status.

To assess tobacco use behaviors and access, we examined several additional variables: frequency of tobacco use (number of days used during the past 30 days), age at first use of tobacco, cessation behaviors (past-year quit attempt and intentions to quit), and source of primary tobacco product (purchased from a store, purchased on the internet, obtained from a friend or family member, or obtained in some other way). Separately, we compared tobacco use behaviors (eg, product use, age at first use, past-year quit attempt) among students who reported current use of menthol and nonmenthol tobacco products. For these comparisons, we defined the nonmenthol group as students who currently used a tobacco product but did not report current menthol tobacco use. Nonmenthol products include both unflavored (tobacco-flavored) and nonmenthol flavored products (eg, candy, fruit, chocolate, mint). We used this binary classification to examine the effects of menthol flavoring.

We suppressed unreliable estimates, defined as an unweighted denominator of less than 50 or a relative SE of more 30%. To account for the complex survey design when comparing groups, we used Rao–Scott χ^2^ tests. To adjust for multiple comparisons, we applied the Benjamini–Hochberg procedure by product type with a false discovery rate of 5%. *P* values of less than .05 were considered significant for all statistical tests. We prioritized descriptive analyses (ie, weighted prevalence estimates and bivariate comparisons) over regression modeling to characterize the prevalence of menthol tobacco use among various groups of students and to highlight subgroups. This approach is well-suited for cross-sectional surveillance data where the primary goal is to identify patterns of menthol tobacco use across diverse products and populations. We conducted all analyses in R version 4.4.2 (R Foundation) with the survey (12) and surveytable (13) packages. This activity was reviewed by the Centers for Disease Control and Prevention, deemed not research, and was conducted consistently with applicable federal law and Centers for Disease Control and Prevention policy.

## Results

### Sociodemographic characteristics and mental health status

During 2023 and 2024, 9.4% of middle and high school students (representing 2.53 million students) reported current tobacco product use. Overall, 1.9% of students (representing 520,000 students) reported current (past 30-day) menthol tobacco product use. Among students who used any tobacco product, 22.1% used menthol tobacco products (Table 1).

**Table 1:** Current (Past 30-Day)^a^ Use of Menthol Tobacco Products Among US High School and Middle School Students Overall and Among Students Who Currently Use Tobacco Products, by Product Type, Sociodemographic Characteristics, and Mental Health Status, National Youth Tobacco Survey, US, 2023–2024

Characteristic	Menthol products^b^		Menthol e-cigarettes^c^		Menthol combustibles^d^		Menthol cigarettes^e^		Menthol cigars^f^	
	Weighted % (95% CI)	No. of students^g^	Weighted % (95% CI)	No. of students^g^	Weighted % (95% CI)	No. of students^g^	Weighted % (95% CI)	No. of students^g^	Weighted % (95% CI)	No. of students^g^
**Overall menthol product use^h^**	1.9 (1.6–2.2)	520,000	1.2 (0.9–1.4)	320,000	0.8 (0.6–0.9)	210,000	0.6 (0.5–0.7)	160,000	0.1 (0.1–0.2)	30,000
**Menthol tobacco use among students who currently used tobacco products**	22.1 (19.2–25.2)	520,000	18.0 (14.8–21.4)	320,000	26.4 (22.2–30.9)	210,000	40.0 (35.1–45.1)	160,000	11.5 (7.8–16.3)	30,000
**Sex**										
Female	18.3^i^ (14.6–22.6)	220,000	14.7^i^ (11.3–18.8)	140,000	23.9 (17.4–31.5)	80,000	37.7 (28.8–47.3)	60,000	9.0 (4.7–15.3)	10,000
Male	26.1 (23.2–29.2)	300,000	22.1 (18.5–26.0)	170,000	28.4 (23.6–33.6)	120,000	41.5 (33.3–50.0)	90,000	13.2 (8.5–19.0)	20,000
**Grade level**										
Middle school (grades 6–8)	17.0^i^ (14.4–19.9)	110,000	10.0^i^ (7.5–13.0)	40,000	25.5 (20.0–31.6)	60,000	41.6 (33.6–50.0)	50,000	^—j^	^—j^
High school (grades 9–12)	23.9 (20.2–27.9)	400,000	20.5 (16.7–24.8)	270,000	26.5 (21.3–32.2)	140,000	39.1 (32.6–45.8)	100,000	10.5 (6.3–16.2)	20,000
**Race and ethnicity**										
Hispanic or Latino	19.5 (16.5–22.8)	140,000	12.6 (9.5–16.2)	60,000	28.1 (23.1–33.5)	70,000	37.0 (29.1–45.4)	50,000	15.3 (8.9–24.0)	10,000
Non-Hispanic American Indian/Alaska Native	22.2 (14.4–31.9)	<10,000	18.5 (9.9–30.2)	<10,000	^—j^	<10,000	^—j^	^—j^	^—j^	^—j^
Non-Hispanic Asian	^—j^	^—j^	^—j^	^—j^	^—j^	^—j^	^—j^	^—j^	^—j^	^—j^
Non-Hispanic Black or African American	12.0 (8.2–16.7)	40,000	9.6 (5.4–15.4)	20,000	11.9 (6.3–20.0)	10,000	^—j^	10,000	^—j^	^—j^
Non-Hispanic Native Hawaiian or Other Pacific Islander	^—j^	^—j^	^—j^	^—j^	^—j^	^—j^	^—j^	^—j^	^—j^	^—j^
Non-Hispanic White	25.8^i^ (21.6–30.3)	260,000	24.0^i^ (19.2–29.3)	190,000	26.6^i^ (20.5–33.4)	80,000	36.6 (28.5–45.4)	60,000	11.0 (6.4–17.4)	10,000
Non-Hispanic multiracial	28.6 (19.8–38.8)	30,000	21.7 (12.6–33.4)	20,000	28.5 (18.2–40.7)	10,000	^—j^	<10,000	^—j^	^—j^
**Overall anxiety and depression, per Patient Health Questionnaire for Anxiety and Depression-4^k^**										
None (0–2 symptoms) or mild (3–5 symptoms)	18.8^i^ (15.7–22.2)	190,000	16.1 (12.4–20.4)	110,000	21.0^i^ (16.1–26.8)	70,000	34.8 (26.2–44.1)	50,000	12.1 (6.5–19.9)	10,000
Moderate (6–8 symptoms) or severe (9–12 symptoms)	25.0 (19.6–31.1)	170,000	21.4 (15.9–27.9)	120,000	28.7 (22.2–35.9)	70,000	45.2 (35.1–55.5)	50,000	8.1 (4.2–13.8)	<10,000

Abbreviation: — , does not apply.

a Current use was defined as using a specified tobacco product for ≥1 day in the past 30 days.

b Menthol product use was assessed for cigarettes, cigars, smokeless tobacco (chewing tobacco, snuff, or dip), e-cigarettes, hookah, pipe tobacco, snus, oral tobacco, bidis, heated tobacco, and nicotine pouches. It does not include roll-your-own cigarettes. Current “any menthol product” use is a composite measure and is defined as currently using ≥1 of the listed products and indicating menthol-flavor use for that product by selecting “menthol” for the question, “In the past 30 days when you used [specific tobacco product], what flavors did you use? (Select one or more)” or if respondents wrote in a response corresponding to “menthol.” Other flavors could also be selected in addition to menthol. Those who selected “some other flavor(s) not listed here” could provide a write-in response. Write-in responses corresponding to an original response option were recoded.

c Current e-cigarette use was categorized as menthol e-cigarette use if respondents selected “menthol” for the question, “In the past 30 days when you used e-cigarettes, what flavors did you use? (Select one or more)” or if they wrote in a response corresponding to “menthol.” Other flavors could also be selected in addition to menthol. Those who selected “some other flavor(s) not listed here” could provide a write-in response. Write-in responses corresponding to an original response option were recoded.

d Combustible products included cigarettes, cigars, hookah, pipe tobacco, or bidis. Current “combustible menthol product” use was defined as currently using ≥1 combustible product and indicating menthol-flavor use for that product. Those who selected “some other flavor(s) not listed here” could provide a write-in response. Write-in responses corresponding to an original response option were recoded.

e Current cigarette use was categorized as menthol cigarette use if respondents answered yes to the question, “Menthol cigarettes are cigarettes that taste like mint. During the past 30 days, were the cigarettes that you usually smoked menthol?”; or if they indicated “Kool” or “Newport” as a brand they usually smoked in the past 30 days. Usual brand was determined based on responses to 2 questions: 1) “During the past 30 days, what brands of cigarettes did you smoke? (Select one or more)” and 2) “During the past 30 days, what brand of cigarettes did you usually smoke? (Choose only one answer).” If “Kool” or “Newport” was the only brand selected for the first question, or if multiple brands were selected in the first question and “Kool” or “Newport” was selected for the second question, “Kool” or “Newport” was considered the respondent’s usual brand. Those who reported no or “not sure” to the menthol question or those who did not report “Newport” or “Kool” as their usual brand were categorized as nonmenthol use; all other respondents reporting past 30-day cigarette use who did not provide any valid responses were assigned as missing menthol smoking status. Those who selected “some other brand(s) not listed here” could provide a write-in response. Write-in responses corresponding to an original response option were recoded.

f Includes cigars, cigarillos, and little cigars. Current cigar use was categorized as menthol cigar use if the student selected “menthol” for the question, “In the past 30 days when you used cigars, cigarillos, or little cigars, what flavors did you use? (Select one or more).”

g Estimated weighted total number of students who reported current use of tobacco products was rounded down to the nearest 10,000 persons. Subgroup estimates might not sum to the overall population estimates because of rounding or exclusion of students who did not report sex, race and ethnicity, or grade level.

h Percentage of all students who indicated menthol tobacco product use for each respective product category, calculated by using responses from students with nonmissing data for questions asking about current use of any tobacco product. These students responded with any value between 0 and 30 for the question, “During the past 30 days, on how many days did you use [tobacco product]”?

i Determined by Rao–Scott χ^2^ test; *P* < .05 considered significant.

j Estimates were statistically unreliable because of an unweighted denominator <50 or a relative SE >30%.

k A brief, 4-item scale used to identify people who may be experiencing anxiety, depression, or both (11). Patient Health Questionnaire for Anxiety and Depression-4 score is determined by 4 questions: “During the past two weeks how often you have been bothered by any of the following problems? 1) little interest or pleasure in doing things; 2) feeling down depressed, or hopeless; 3) feeling nervous, anxious, or on edge; and 4) not being able to stop or control worrying.” Response options include a 4-level ranking of 1) not at all, 2) several days, 3) more than half of the days, or 4) nearly every day. Scores were assigned by response as follows: 0 = not at all, 1 = several days, 2 = more than half of the days, and 3 = nearly every day. Score totals were added for each of the 4 questions. A total score of 0–2 = normal, 3–5 = mild, 6–8 = moderate, and 9–12 = severe anxiety or depression. Only respondents who answered all 4 questions were included in Patient Health Questionnaire for Anxiety and Depression-4 score calculations.

Among students who currently used any tobacco product, the prevalence of menthol tobacco product use was higher among males than females (26.1% vs 18.3%; *P* < .001). The proportion who reported menthol tobacco use was higher among high school students than among middle school students (23.9% vs 17.0%; *P* = .002). We also found differences by race and ethnicity, with use ranging from 12.0% among non-Hispanic Black students to 28.6% among non-Hispanic multiracial students. The proportion who used menthol products was higher among students who reported symptoms of moderate to severe anxiety or depression compared with the proportion among students with no or mild symptoms of anxiety or depression (25.0% vs 18.8%; *P* = .03).

Among students who currently used e-cigarettes, 18.0% reported using menthol e-cigarettes. A higher proportion of males used menthol e-cigarettes compared with females (22.1% vs 14.7%; *P *= .002), and a higher proportion of high school students used menthol e-cigarettes compared with middle school students (20.5% vs 10.0%; *P *< .001). Use of menthol e-cigarettes differed among racial and ethnic groups and ranged from 9.6% of non-Hispanic Black students to 24.0% of non-Hispanic White students. We observed no significant difference by mental health status in the use of menthol e-cigarettes.

Among students who used any combustible product, 26.4% reported using at least 1 menthol combustible product. By race and ethnicity, use ranged from 11.9% among non-Hispanic Black students to 28.5% among non-Hispanic multiracial students. Among students with symptoms of moderate or severe anxiety or depression, 28.7% used menthol combustible products compared with 21.0% of students with no or mild symptoms of anxiety or depression (*P* = .04). Among students who reported current cigarette use, 40.0% reported smoking menthol cigarettes, and among students who reported current cigar use, 11.5% reported smoking menthol cigars; we found no significant differences in use of menthol cigarettes or cigars by sociodemographic characteristics or mental health status.

### Tobacco use behaviors by menthol and nonmenthol product use

Among students who used both e-cigarettes and cigarettes, a higher proportion of students who used menthol e-cigarettes also used menthol cigarettes compared with those who used nonmenthol e-cigarettes (63.9% vs 35.7%; adjusted *P* < .001) (Table 2). About one-quarter of students (24.3%) who reported menthol e-cigarette use also reported menthol cigar use; similarly, 25.7% of those who used menthol cigarettes also reported menthol cigar use.

**Table 2:** Tobacco-Related Behaviors by Current (Past 30-Day)^a^ Use of Menthol and Nonmenthol Tobacco Products Among US High School and Middle School Students Who Currently Use Tobacco Products, National Youth Tobacco Survey, US, 2023–2024

Behavior	E-cigarettes			Cigarettes			Cigars, cigarillos, and little cigars		
	Menthol,^b^ weighted % (95% CI)	Nonmenthol,^c^ weighted % (95% CI)	Adjusted *P* value^d^	Menthol,^e^ weighted % (95% CI)	Nonmenthol,^f^ weighted % (95% CI)	Adjusted *P* value^d^	Menthol,^g^ weighted % (95% CI)	Nonmenthol,^h^ weighted % (95% CI)	Adjusted *P* value^d^
**Use of other menthol tobacco products**									
Menthol e-cigarettes	NA	NA	NA	51.6 (39.3–63.9)	25.1 (18.8–32.2)	<.001^i^	—j	23.0 (15.9–31.4)	—j
Menthol cigarettes	63.9 (52.4–74.4)	35.7 (28.7–43.2)	<.001^i^	NA	NA	NA	—j	51.6 (42.2–61.0)	—j
Menthol cigars	24.3 (15.7–34.8)	—j	—j	25.7 (17.0–36.0)	—j	—j	NA	NA	NA
**Frequency of tobacco product use**									
1–5 Days in the last 30 days	24.8 (19.2–31.1)	49.1 (44.3–54.0)	<.001^i^	50.0 (39.4–60.6)	66.1 (56.7–74.7)	.02^i^	33.3 (17.7–52.2)	58.3 (52.1–64.3)	.03^i^
6–19 Days in the last 30 days	21.7 (12.2–34.2)	17.7 (15.3–20.4)	.47	11.9 (7.5–17.7)	13.6 (9.4–18.9)	.66	25.3 (7.2–53.4)	19.1 (14.3–24.7)	.55
20–30 Days in the last 30 days	53.4 (43.9–62.7)	33.2 (28.6–38.0)	<.001^i^	38.2 (28.9–48.0)	20.2 (14.1–27.6)	.002^i^	41.3 (22.8–61.9)	22.5 (17.2–28.6)	.05
**Age at first use of tobacco**									
Elementary school aged (8–10 y)	19.9 (14.9–25.8)	13.2 (10.6–16.1)	.02^i^	42.5 (33.8–51.5)	26.8 (21.5–32.5)	.004^i^	—j	19.1 (14.8–24.1)	—j
Middle school aged (11–13 y)	39.3 (31.7–47.4)	42.3 (38.5–46.2)	.45	24.7 (18.5–31.7)	30.5 (24.1–37.5)	.37	—j	33.4 (25.4–42.2)	—j
High school aged (≥14 y)	40.7 (31.7–50.3)	44.5 (40.2–48.8)	.45	32.8 (22.4–44.6)	42.7 (34.7–51.1)	.17	—j	47.4 (39.7–55.3)	—j
**Mental health indicator**									
Initiated e-cigarette use because they were feeling anxious, stressed, or depressed^k^	42.5 (37.4–47.7)	28.6 (24.5–33.0)	<.001^i^	NA	NA	NA	NA	NA	NA
**Cessation behaviors**									
Past-year quit attempt^l^	63.6 (56.6–70.3)	71.4 (67.8–74.8)	.06	49.7 (40.5–59.0)	63.2 (57.5–68.6)	.04^i^	NA	NA	NA
Intentions to quit^m^	68.1 (60.8–74.7)	74.0 (70.3–77.4)	.17	63.4 (52.8–73.2)	67.2 (60.6–73.3)	.56	NA	NA	NA
**Source of primary tobacco product used**									
Bought products from a store^n^	37.8 (30.0–46.0)	20.1 (17.1–23.2)	<.001^i^	—j	—j	—j	—j	23.9 (17.6–31.3)	—j
Purchased products on the internet^o^	7.2 (4.7–10.6)	2.4 (1.4–3.8)	.003^i^	6.1 (3.2–10.4)	—j	—j	—j	^—j^	—j
Obtained products from a friend or family member^p^	42.5 (35.3–50.1)	35.5 (32.2–38.9)	.09	36.4 (27.3–46.2)	28.2 (22.5–34.5)	.19	—j	29.8 (23.7–36.6)	—j
Obtained products in some other way^q^	61.9 (55.3–68.2)	60.7 (57.0–64.3)	.74	59.6 (49.0–69.6)	63.9 (55.4–71.8)	.49	75.8 (59.0–88.2)	62.3 (55.6–68.7)	.18

Abbreviation: NA, not applicable.

a Current (past 30-day use) was defined as using a specified tobacco product for ≥1 day in the past 30 days.

b Current e-cigarette use was categorized as menthol e-cigarette use if respondents selected “menthol” for the question, “In the past 30 days when you used e-cigarettes, what flavors did you use? (Select one or more)” or if they wrote in a response corresponding to “menthol.” Other flavors could also be selected in addition to menthol. Those who selected “some other flavor(s) not listed here” could provide a write-in response. Write-in responses corresponding to an original response option were recoded.

c Current e-cigarette use was categorized as nonmenthol e-cigarette use if respondents selected any flavor other than “menthol” for the question, “In the past 30 days when you used e-cigarettes, what flavors did you use? (Select one or more).” This categorization includes students who used the following flavors: tobacco-flavored; mint; spice (such as cinnamon, vanilla, or clove); fruit, chocolate; alcoholic drinks (such as wine, margarita, or other cocktails); nonalcoholic drink (such as coffee, soda, lemonade, or other beverage); candy, desserts, or other sweets; or unflavored.

d Adjusted Rao–Scott χ^2^ *P* values compared prevalence of selected characteristics between respondents who reported use of menthol products with respondents who reported use of nonmenthol products. *P* values were adjusted by using the Benjamini–Hochberg procedure to control for multiple comparisons by product type.

e Current cigarette use was categorized as menthol cigarette use if respondents answered yes to the question, “Menthol cigarettes are cigarettes that taste like mint. During the past 30 days, were the cigarettes that you usually smoked menthol?”; or if they indicated “Kool” or “Newport” as a brand they usually smoked in the past 30 days. Usual brand was determined based on responses to 2 questions: 1) “During the past 30 days, what brands of cigarettes did you smoke? (Select one or more)” and 2) “During the past 30 days, what brand of cigarettes did you usually smoke? (Choose only one answer).” If “Kool” or “Newport” was the only brand selected for the first question, or if multiple brands were selected in the first question and “Kool” or “Newport” was selected for the second question, “Kool” or “Newport” was considered the respondent’s usual brand. Those who selected “some other brand(s) not listed here” could provide a write-in response. Write-in responses corresponding to an original response option were recoded.

f Those who reported no or “not sure” to the menthol question or those who did not report “Newport” or “Kool” as their usual brand were categorized as nonmenthol use; all other respondents reporting past 30-day cigarette use who did not provide any valid responses were assigned as missing menthol smoking status.

g Current cigar use was categorized as menthol cigar use if they selected “menthol” for the question, “In the past 30 days when you used cigars, cigarillos, or little cigars, what flavors did you use? (Select one or more).”

h Current cigar use was categorized as nonmenthol cigar use if respondents selected any flavor other than “menthol” for the question, “In the past 30 days when you used cigars, cigarillos, or little cigars, what flavors did you use? (Select one or more).” This categorization includes students who used the following flavors: tobacco-flavored; mint; spice (such as cinnamon, vanilla, or clove); fruit, chocolate; alcoholic drinks (such as wine, margarita, or other cocktails); nonalcoholic drink (such as coffee, soda, lemonade, or other beverage); or candy, desserts, or other sweets.

i Rao-Scott χ^2^ test considered significant after Benjamini–Hochberg procedure with false discovery rate of 0.05.

j Estimates were statistically unreliable because of an unweighted denominator <50 or a relative SE >30%.

k Respondent indicated “I was feeling anxious, stressed, or depressed” when asked “Why did you first use an e-cigarette? (Select one or more).”

l Defined as indicating ≥1 time to the question “During the past 12 months, how many times have you stopped using [tobacco product] for one day or longer because you were trying to quit using [tobacco product] for good?”

m Defined as responding yes to the question “Are you seriously thinking about quitting [tobacco product]? (Please choose the first answer that fits).”

n Defined as responding “I bought them myself” to the question “During the past 30 days, how did you get your [tobacco product]? (Select one or more)” and with “gas station or convenience store,” “grocery store,” “drugstore,” “mall or shopping center kiosk/stand,” “vending machine,” or “vape shop or tobacco shop” to the question “During the past 30 days, where did you buy your [tobacco product]? (Select one or more).”

o Defined as responding “I bought them myself” to the question “During the past 30 days, how did you get your [tobacco product]? (Select one or more)” and “On the internet (such as a product website, online vape or tobacco store or other online marketplace” or “Through a delivery service (such as DoorDash or Postmates)” to the question “During the past 30 days, where did you buy your [tobacco product]? (Select one or more).”

p Defined as responding “I got them from a friend” or “I got them from a family member” to the question “During the past 30 days, how did you get your [tobacco products]? (Select one or more).”

q Defined as responding, “I had someone else buy them for me,” “I asked someone to give me some,” “Someone offered them to me,” “I took them from a store or another person,” or “I got them in some other way” to the question “During the past 30 days, how did you get your [tobacco products]? (Select one or more).”

Among students who used menthol e-cigarettes, 53.4% reported use on 20 to 30 of the last 30 days, while among students who used nonmenthol e-cigarettes, 33.2% reported use on 20 to 30 of the last 30 days (adjusted *P* < .001). Similarly, among students who smoked menthol cigarettes, 38.2% reported use on 20 to 30 of the last 30 days, while among students who used nonmenthol cigarettes, 20.2% reported use on 20 to 30 of the last 30 days (adjusted *P* = .002). We found a similar pattern among students who smoked menthol (vs nonmenthol) cigars (41.3% vs 22.5%; adjusted *P* = .05).

Among students who currently smoked cigarettes, the prevalence of first trying a cigarette at an age of 8 to 10 years was 42.5% for those who currently smoked menthol cigarettes and 26.8% for those who currently smoked nonmenthol cigarettes (adjusted *P* = .004). Similarly, among students who currently used e-cigarettes, the proportion who first tried an e-cigarette at an age of 8 to 10 years was 19.9% among those who currently used menthol e-cigarettes and 13.2% among those who currently used nonmenthol e-cigarettes (adjusted *P* = .02). The prevalence of reporting feeling anxious, stressed, or depressed as a reason for trying the tobacco product was 42.5% among students who currently used menthol e-cigarettes and 28.6% among students who currently used nonmenthol e-cigarettes (adjusted *P* < .001).

The proportion of students who reported a past-year cigarette quit attempt was lower among students who currently smoked menthol cigarettes (49.7%) than among those who currently smoked nonmenthol cigarettes (63.2%) (adjusted *P* = .04). Among students who used menthol e-cigarettes, 63.6% reported a past-year e-cigarette quit attempt compared with 71.4% of students who used nonmenthol e-cigarettes (adjusted *P* = .06). We found no significant differences across tobacco products in reported intention to quit by menthol versus nonmenthol tobacco use.

The proportion of students who bought their products from a store or on the internet was higher among students who currently used menthol e-cigarettes than among students who currently used nonmenthol e-cigarettes (37.8% vs 20.1%, adjusted *P* < .001 and 7.2% vs 2.4%, adjusted *P* = .003, respectively). Obtaining products from a friend or family member or in some other way did not differ significantly by menthol versus nonmenthol tobacco use.

## Discussion

In 2023 and 2024, more than a half-million US middle and high school students reported current menthol tobacco product use. Prevalence varied by sex, race and ethnicity, grade level, and mental health status. Among students who currently used e-cigarettes, those who used menthol (vs nonmenthol) e-cigarettes differed in their reported age of first use of tobacco, reasons for first use, frequency of product use, and where products were acquired. Students who used menthol (vs nonmenthol) cigarettes smoked cigarettes at a higher frequency, started smoking at a younger age, and were less likely to have made a quit attempt in the past year. Students who used menthol cigars smoked cigars more frequently than students who smoked nonmenthol cigars.

The prevalence of using any menthol tobacco product or menthol e-cigarettes was higher among male students than among female students. A systematic review of literature published from 2011 through 2017 found that females were more likely to smoke menthol cigarettes than males and had a greater prevalence of menthol tobacco use among both adolescent and adult populations (14). This discrepancy suggests that sex differences in menthol tobacco use among young people may be shifting. However, it is important to note that this shift may reflect the evolving landscape of the tobacco market: the wide availability of diverse nonmenthol flavors in products like e-cigarettes, compared with combustible cigarettes, may appeal differently to male and female children, adolescents, and young adults.

Among students who currently used e-cigarettes, a higher proportion of those using menthol products, compared with those using nonmenthol products, reported that they first used an e-cigarette to cope with feelings of anxiety, stress, or depression. Additionally, current menthol tobacco use was higher among those who reported symptoms of moderate or severe anxiety or depression compared with those reporting no or mild symptoms of depression or anxiety. Young people may use e-cigarettes to relieve symptoms of depression and anxiety; however, nicotine addiction and withdrawal can contribute to or worsen these symptoms (15). While our study population was middle and high school students, our findings are consistent with another study showing a link between menthol tobacco use and anxiety and depression in young adult populations (16). This link was pronounced in our study among students currently using menthol tobacco; however, all young people who use tobacco products to cope with mental health stressors may benefit from integrated public health interventions that combine tobacco cessation support with evidence-based mental health resources and development of healthy coping skills. Nicotine is a driving factor for tobacco use, and menthol makes it easier to initiate and continue smoking. Menthol acts as a cue and reinforcer of nicotine uptake, making tobacco products even more addictive (2,17). In our study, students who currently used menthol e-cigarettes reported a higher frequency of use than those who used nonmenthol e-cigarettes. Students who smoked menthol cigarettes reported higher frequency of use, and a lower proportion of these students, compared with those who smoked nonmenthol cigarettes, reported a past-year quit attempt. These findings contribute to literature suggesting that menthol tobacco use may lead to increases in initiation and continued use of tobacco products (2,17).

Preventing children, adolescents, and young adults from initiating tobacco product use and supporting cessation efforts can reduce nicotine dependence and long-term health risks (18). Our study has important public health implications. Specifically, the higher frequency of use and younger age of initiation among young people who use menthol products underscore the potential public health impact of comprehensive public health efforts and strategies. Comprehensive strategies include strengthening licensing requirements for brick-and-mortar and online tobacco retailers to limit access; raising prices, including through state excise taxes, minimum prices, and bans on discounting; and eliminating marketing focused on young people (2,19). These strategies may be especially useful considering that a higher percentage of students who currently used menthol e-cigarettes (vs nonmenthol e-cigarettes) obtained them from stores or online. Furthermore, educational programs that emphasize the harm of tobacco products, including menthol products, may help increase understanding among young people of the dangers of tobacco and nicotine use.

### Limitations

This study has several limitations. First, self-reported data are subject to social desirability and recall bias; however, reporting use of menthol versus nonmenthol products is unlikely to be affected by these biases. Second, NYTS was completed by students in public and private schools; therefore, the findings might not be generalizable to young people who are home-schooled, have dropped out of school, or are in detention centers or other settings (8–10). Third, students may have trouble distinguishing between menthol and mint-flavored tobacco products. However, previous NYTS cognitive testing (unpublished) found that most students accurately distinguish these flavors. Fourth, the relatively low survey response rates for the 2023 and 2024 NYTS can increase the potential for nonresponse bias, resulting in higher SEs for some estimates. Higher SEs can reduce the power to detect significant differences. However, NYTS survey weights are adjusted each survey cycle to reduce the potential for nonresponse bias. Lastly, because NYTS is a cross-sectional survey, menthol tobacco use status at the time of tobacco use initiation and at the time of past-year cessation attempt cannot be determined.

### Conclusion

More than 1 in 5 middle and high school students who use tobacco products use menthol, with differences by sociodemographic characteristics, mental health status, and tobacco product. Our findings add to the growing body of literature highlighting the role of menthol tobacco among children, adolescents, and young adults who use cigarettes or e-cigarettes and factors such as younger age of initiation, reasons for first tobacco use, higher frequency of use, and fewer cessation attempts among young people who smoke menthol cigarettes. Our findings suggest that further addressing access to menthol tobacco products, alongside mental health support, may be important components of broader public health strategies to reduce tobacco use in this population.
